# Neck and Upper Extremity Musculoskeletal Symptoms Secondary to Maladaptive Postures Caused by Cell Phones and Backpacks in School-Aged Children and Adolescents

**DOI:** 10.3390/healthcare11060819

**Published:** 2023-03-10

**Authors:** Danny G. Warda, Uzoma Nwakibu, Ali Nourbakhsh

**Affiliations:** Department of Orthopedic Surgery, Kennestone Hospital, Marietta, GA 30060, USA; danny.warda@wellstar.org (D.G.W.); uzoma.nwakibu@wellstar.org (U.N.)

**Keywords:** text neck, cell phone, backpacks, musculoskeletal symptoms, maladaptive postures, school aged children, cervical spine, upper extremity

## Abstract

Technology is an essential part of our lives. Nowadays, it is almost impossible to leave the house without a cell phone. Despite the wide range of benefits of cell phones and handheld electronic devices, this evolution of technology has not come without a price. The pandemic of cell phone use among children and young adolescents has led to the emergence of a set of musculoskeletal (MSK) symptoms that have not been seen before in this age group. These symptoms can range from neck and shoulder discomfort to pain, peripheral neurological symptoms of the upper extremity, and long-term complications such as disk prolapse and degenerative disk disease of the cervical spine. This clinical presentation is known as “text neck syndrome.” In addition to MSK symptoms, text neck syndrome could also include eye and ear symptoms, psychological problems, peripheral neurological symptoms, and poor academic performance. Multiple mechanisms have been discussed by which cell phone use causes MSK symptoms. Maladaptive postures, a decrease in physical activity leading to obesity, and the direct effect of electromagnetic radiation are some of the mechanisms by which long-term use of cell phones leads to the clinical presentation of text neck syndrome and its long-term consequences. The purpose of this article is to review the literature, discuss the epidemiology of cell phone use and MSK symptoms associated with its use in children and adolescents, describe its clinical presentation, explain the pathophysiology behind it, and provide preventative guidelines that can be used by this age group to allow for the continued use of these electronics without harmful effects on their posture and long-term health.

## 1. Introduction

PubMed was searched for articles relevant to neck and upper extremity musculoskeletal symptoms secondary to maladaptive postures caused by cell phones and backpacks in school-aged children and adolescents. “Cervical spine and cell phones,” “cell phones and spine,” “children’s spine and smartphones,” “smartphones and cervical spine,” “cell phones and neuropathy,” “peripheral neuropathy in peds,” and “peripheral neuropathy and cell phone” were the specified phrases used in our search. Results were limited to English-language abstracts, but no date limitations were used. All abstracts were reviewed for relevance, and relevant papers were further examined for relevance to the children and adolescent age group. The bibliographies of the included articles were searched for additional articles not included in the database search. We found a total of 79 articles on the topic of our interest.

Technology is an essential part of our lives. Nowadays, it is impossible to leave the house without a cell phone. The evolution of handheld electronics and cell phone technology and the wide range of benefits they offer have not come without a price. The widespread use of cell phones and their effect on health is currently a hot topic in the literature [1,2,3,4,5,6,7,8,9,10,11,12,13,14,15,16,17,18,19,20,21,22,23,24,25,26,27,28,29,30,31,32,33,34,35]. Of special interest to us is the long-term effect of their use on children and adolescents. The extended hours of desktop use in adults in work environments has been linked to the development of maladaptive posture called forward head posture, which in turn leads to musculoskeletal (MSK) pain [1,24,36,37,38,39,40]. The pandemic of cell phones and handheld electronic devices and their widespread use among school aged children and adolescents has caused this age group to develop a constellation of musculoskeletal symptoms collectively called “text neck syndrome” [1,3,17,18,25,33,41]. The clinical presentation of text neck syndrome varies from neck and shoulder discomfort to headache, pain in the neck, shoulder and upper extremities, eye and ear symptoms, psychological problems, peripheral neurological symptoms, and poor academic performance [1,27,28,29,30,31,32,41,42,43,44,45,46,47,48,49].

Multiple mechanisms have been discussed by which cell phone use causes MSK symptoms; simply, it starts with spending large portions of the day with the neck flexed looking down at these devices, often for hours at a time. This leads to altered posture of the spine, which in turn affects the surrounding muscles and ligaments, leading to weakness of the deep muscles of the spine and structural changes in the ligaments, putting more pressure on the erector spinae muscles to maintain stability of the spine and counteract those changes, leading to more strain and eventually fatigue, and the end results are pain and instability of the spine, putting it at risk of degenerative changes and disk herniation in the future [3,4,15,16,17,18,20,21,22,23,24,25,36,37,39,50,51,52,53,54,55]. The electromagnetic effect, obesity resulting from a sedentary lifestyle, and the added load of a backpack on children are other factors that also play a role in the worsening of the musculoskeletal symptoms [1,26,49,55,56].

Prevention is the answer to this pandemic. The purpose of this article is to review the literature, discuss the epidemiology of cell phone use and musculoskeletal symptoms in children and adolescents, list the signs and symptoms of text neck syndrome, explain the mechanism by which these symptoms are caused, increase the awareness of physicians and the general public to these problems, and provide some prevention guidelines that can be used early to guide this age group to adapt new ways of using these handheld devices that will enable them to still take advantage of their benefits without having a harmful effect on their posture and hopefully prevent the long-term health problems associated with their use.

## 2. Epidemiology

### 2.1. Cell Phone Use

It is estimated that 75% of the population spends several hours every day with their heads bent forward, looking down at their mobile devices [1,40]. Cell phone use is more common in adults; about 72% of adults in the USA own an electronic gadget [2,3], while 95% of homes with kids have a smartphone and 78% have a tablet. Furthermore, 42% of children have a tablet, which can be used for a myriad of activities, such as watching YouTube videos, playing video games, watching TV shows or movies, and reading books [1,57].

Duration of use is an important factor in putting the person at risk of developing MSK symptoms. Prolonged hours of use are common in all age groups. Cell phones are used for about 5.1 h a day by adults and for more than 8.5 h a day by college students (19–22 years old) [3,4,5]. According to a Swiss study, more than 50% of adult participants spend about 2 h of their day on cell phones [6,7,8]. Based on a media survey of 13–18-year-old children, 46% of their time spent on electronics was on cell phones and handheld devices, and 8–12-year-old children spend 41% of their screen time on cell phones and handheld devices [3,58].

### 2.2. Musculoskeletal Neck Pain

Neck pain has an incidence of 40% and is the eighth leading cause of years lived with disability in the 15–19-year-old age group [1,10,59]. Having neck pain early in life is a risk factor and a great predictor for developing further episodes in the future and chronic pain as adults [1,12,40,60,61].

### 2.3. Gender Differences

Text neck posture has equal prevalence in both genders [3,13]. However, men have demonstrated higher flexion angles of the head and neck when using handheld devices, which might place them at increased risk of the long-term consequences of this maladaptive posture [3,13,62]. Even though males showed higher flexion angles compared to females, musculoskeletal symptoms of the neck and upper extremities secondary to text neck posture had higher prevalence in females in one of the studies [1,3,63,64].

## 3. Pathophysiology and Risk Factors

### 3.1. Altered Posture

Two maladaptive postures are described in the literature: the forward head posture and the text neck posture. Forward head posture is characterized by lower cervical flexion and higher cervical extension and is caused by activities that require forward shifting of the head and maintaining the eyes at horizontal level, as often seen among those using computers, handheld devices, or carrying backpacks [3,36,37]. Text neck posture, on the other hand, is characterized by generalized excessive flexion of the whole cervical spine without upper cervical extension and is caused by activities that force us to look down lower than eye level, such as texting [3].

The consequences of these maladaptive postures are anatomical and physiological changes that might explain the clinical presentation. Forward neck posture affects the cervical and thoracic spine, leading to decreased cervical lordosis and increased thoracic kyphosis. This ultimately causes stretching of the anterior cervical and posterior thoracic muscles and ligaments and shortening of the posterior cervical and anterior thoracic muscles and ligaments. Additionally, there is resultant protraction and medial rotation of the scapula [3,4,36,37,50,51]. Long-term flexed posture of the cervical spine leads to a vicious cycle of gradual muscle weakness and increased stress on the ligamentous structures, which causes them to creep, and subsequent worsening of the posture. In order to compensate, the erector spinae muscles are required to work harder to overcome all those changes, leading to their fatigue and further worsening of the posture [3,4,37,50,51,52]. Other consequences of this vicious cycle are spinal instability and disk prolapse [3]. Text neck posture leads to many similar changes to those observed to be caused by forward neck posture; the only difference is stretching of the posterior cervical muscle instead of shortening, which leads to widening of the posterior space between the occiput, C1, and C2, and stretching of ligaments in the area, leading to upper cervical instability, putting the spinal cord, vertebral artery, and greater occipital nerve at risk of injury [3,15]. A case of progressive compression myelopathy has been reported in a 38-year-old, previously healthy man who slept with his neck flexed while using his cell phone on a couch with two thick pillows behind his back [16].

Cell phone users tend to hold their necks at 45 degrees of flexion [15,17]. Higher degrees of flexion are observed with cell phone use compared to web browsing and cell phone use while standing compared to sitting [15,17,18]. The weight of the head and thereby the load exerted on the cervical spine increase with increasing flexion angles. The weight of the head ranges from 10 to 12 lbs at neutral to 27 lbs at 15 degrees, 40 lbs at 30 degrees, 49 lbs at 45 degrees, and 60 lbs at 60 degrees [17,53]. In examining the effect of a work environment that requires the use of cell phones on 40 young individuals, two-thirds developed neck and upper back pain that was described as aching after 30 min of cell phone use with a flexed neck [19]. Supported back and forearms and a neutral neck are less likely to be associated with musculoskeletal symptoms [17,20]. Increased disk pressure is a proven risk factor for lumbar spine degenerative disk disease. With the same mechanism, high loads and stresses exerted on the cervical spine by maladaptive postures might lead to increased disk pressure and eventually cervical spine degenerative disk disease [17,54]. In a study of 2438 young individuals with persistent neck discomfort, MRI was used to evaluate the association between the degree of cervical disc degeneration using a specific cervical disk degeneration scale (CDDS) and smartphone use. A total of 52.9% of patients were classified as excessive smartphone users based on a specific scale they used in the study called the smartphone addiction scale. Patients who used their smartphones extensively had higher CDDS scores than those who did not [21]. Another factor that might contribute to cervical spine degeneration is a lordotic angle of less than 20–30 degrees [22] Smartphone use for longer than three hours has a greater impact on spinal degeneration parameters (degenerative disk severity score, disc placement, and modic changes) than smartphone use for less than three hours [23]. Impaired proprioception and plastic changes in the CNS leading to sensory impairment are other long-term consequences of these postures [3,24,38]. A study that compared proprioception after 40 min of cell phone use in a group of healthy individuals found a significant difference between the group that maintained a normal head position, defined as a craniovertebral angle (CVA) of more than 50 degrees, and the other group with a CVA of less than 49 degrees. Each participant wore a headband with a laser pointer to measure cervical proprioception. The participant stood 90 cm from the wall and focused on a target. After closing their eyes, the evaluator rotated the participant’s head left or right, and then instructed them to return to the center. A score was given based on the distance from the center. There was a significant difference in scores between the groups [39].

### 3.2. Duration of Time Spent in Flexed Position

There is a strong, linear association between neck and upper back pain and time spent texting [11,17]. According to a questionnaire given to 522 college students in Brazil, an association was found between time spent on a cell phone, texting style, posture, and neck pain. The longer the time spent on a cell phone and the greater the flexion angle, the greater the likelihood of experiencing neck pain [25]. Children and adolescents spend about 5–7 h a day with their neck in a flexed forward position while using their cell phone, which is about 1825 to 2555 h a year [1,53]. The long hours spent on handheld devices correlate with a sedentary lifestyle and lack of physical activity, which has been linked to an increased susceptibility to having musculoskeletal symptoms. According to a three-year study, teenagers who engaged in regular physical activity were much less likely to experience back discomfort [1,65].

### 3.3. Electromagnetic Field

Smartphones, laptops, wireless internet, and televisions emit an extremely low-frequency electromagnetic field [1,49]. Electromagnetic radiation has been associated with multiple psychological, musculoskeletal, and neurological disorders in children. This is a very important factor to consider, especially in children, because compared to adults, the absorption of electromagnetic fields in children is greater than two-fold in the head and greater than ten-fold in the bone marrow of the skull, which makes them more susceptible to its harmful effect [1,26,48].

### 3.4. Backpacks

Backpack loads can have a harmful effect on the cervical and thoracic spine as well. The added posterior weight of a heavy backpack causes postural alterations that lead to cervical and thoracic spine abnormalities. These postural alterations include a forward trunk lean and a forward head position. When combined with the added cognitive and visual demands of a handheld device, the resultant cervical spine posture may be harmful. Many studies have examined the influence of backpack weights on posture and concluded that people should carry no more than 10% of their body weight in their backpacks. Even carrying 5–10% of adolescent BW causes undesirable postural compensations, including forward head posture, rounded shoulders, a forward trunk lean, and increased lumbar lordosis [66,67,68,69].

### 3.5. Obesity

Sedentary lifestyles and a lack of physical activity are associated with the prolonged use of handheld electronics, leading to weight gain [1,32,70]. In Canada, for example, less than 10% of kids are achieving the recommended daily amount of moderate to vigorous physical activity (MVPA) [1,71]. A relationship has been shown between the amount of adipose tissue in obesity and asymmetry in the scapular area [1,55]. The incidence of childhood obesity is increasing, and according to the World Health Organization, it is considered one of the top ten health issues in childhood [1]. Children are at increased risk of postural problems secondary to this increase in the prevalence of obesity [72].

## 4. Clinical Features

The clinical presentation of text neck posture is called text neck syndrome. It should be considered in children and adolescents who have musculoskeletal symptoms but no underlying musculoskeletal issues. Text neck syndrome has a wide range of clinical presentations, ranging from pain and discomfort of the neck, upper back, and upper extremities to presenting with psychological symptoms, eye symptoms, or symptoms of degenerative disk disease.

### 4.1. Chronic Musculoskeletal Pain of the Neck and Upper Extremities without Neurological Deficit

According to a study of 180 patients between 8 and 17 years old who spent 5–7 h a day using their cell phone with their neck flexed about 45 degrees, the pain was most common in the neck (100% of the sample), followed by the shoulders (69%), lower back (61%), and arms (13%), and lasted more than 6 months [1,41].

### 4.2. Headache

There is an established relationship between headaches and shoulder pain [1,42,43]. Musculoskeletal pain may lead to muscle tension in the neck, shoulders, scalp, or jaw, which in turn might cause headaches [1]. A survey of Swedish schoolchildren revealed an incidence of headaches of 48.0% [1,44,45]. It is also the most prevalent symptom among females (42%) [1,46].

### 4.3. Eye Symptoms

From most common to least common, some of the eye symptoms are eye strain, dry eye, and nearsightedness [1,41,47].

### 4.4. Effect of Electromagnetic Field

Sleep disturbance, behavioral problems, peripheral neurological symptoms such as tingling hands, headaches, dizziness, cardiovascular problems, weakened immunity, vision, and hearing issues have all been linked to electromagnetic radiation [1,48].

### 4.5. Psychological Symptoms

The incidence of mental health problems is higher in kids who spend more than 2 h on electronics [1,27]. Irritability, poor communication, isolation, stress, anxiety, and poor school performance have been associated with the prolonged use of handheld electronics [1,48]. The longer the duration of time spent on such devices, the higher the likelihood of these psychological issues [1,28].

### 4.6. Peripheral Compressive Neuropathy

According to a case–control study that compared 95 cases of clinically diagnosed carpal tunnel syndrome (CTS) confirmed with electromyography to 190 controls with no signs of CTS, it was found that the prevalence of CTS is substantially correlated with daily smartphone use for at least two hours. A total of 4 h or more of daily smartphone use is linked to CTS after controlling for confounders. Furthermore, compared to people who used their smartphone in one hand, those who used both hands had a 7.8-fold greater risk of acquiring CTS [29].

In a study that compared the conduction velocity of the ulnar nerve across the elbow after 6, 9, 12, 15, and 18 min of phone use between a group of patients with pure ulnar sensory symptoms and another group free of symptoms [30], prolonged phone use was also linked to changes in ulnar nerve conduction.

### 4.7. Others

As discussed earlier, thoracic kyphosis increases with these maladaptive postures, and since hyperkyphosis has been identified as a factor that leads to cardiovascular and pulmonary issues, cell phone use could also be to blame. In fact, taking a breath is much harder with these maladaptive postures because of the forward flexion at the waist and rounding of the shoulders [1,31]. Weight gain because of a sedentary lifestyle and lack of physical activity is associated with the prolonged use of handheld electronics [1,32,70].

## 5. Prevention

A variety of factors put school-aged children at risk of adopting abnormal postures, which increases the prevalence of musculoskeletal symptoms and other manifestations of text neck syndrome. Many of these factors were once only applicable to adults, such as the use of desktop computers and cell phones; however, as technology has advanced, these devices have become more commonplace in the everyday lives of growing children. Even school itself can be a contributing factor since children spend significant parts of their days at school sitting at a desk [1]. Sedentary lifestyles, a lack of physical activity, and eventual obesity are other consequences of these devices, which worsen musculoskeletal symptoms [1,71,73,74,75]. These maladaptive postures not only cause symptoms in childhood but also put those kids at higher risk of problems as adults [1,11,12,60,61]. Therefore, the best way to approach this problem is through prevention. Educating the public about the musculoskeletal symptoms of the neck and upper extremities in school-aged children is the best place to start. Understanding the behaviors and risk factors that alter children’s posture and put them at risk of developing those symptoms is key to providing preventative guidelines to allow them to take advantage of the benefits handheld electronic devices provide without putting their current and future health at risk.

Prevention guidelines should be directed to all age groups, including children, adolescents, and their parents [33,34,35]. The goals of these guidelines are aimed at encouraging physical activity and avoiding excessive and prolonged neck flexion [76,77,78]. In addition, they should provide the parents with strategies that enable them to build an environment that encourages these guidelines [35]. Several guidelines have been referenced in the literature [17,33,34,35].

The following efforts should be taken when using cell phones or other handheld devices [1,17,33]: Hold the cell phone at the same level as the eyes to prevent neck flexion and ease pressure on the shoulders and spine; use both hands to hold the cell phone and both thumbs for texting; stretch the anterior cervical tissue and strengthen the posterior muscles of the neck and upper back; avoid overdoing these activities, and remember to take frequent rests, avoid staying in one place and the same position for too long; try to stay away from activities that involve a lot of repetitive motion, such as typing or swiping for lengthy periods of time; and avoid using devices that are too big or too heavy to handle in one hand for extended periods of time.

When it comes to the duration of time spent on handheld devices or screen time, the Australian Department of Health developed the following guidelines in 2012: Children younger than two years should not be exposed to electronic media at all during their waking hours, and screen time should not exceed one hour for children aged 2–5 years or two hours per day for children and young adolescents aged 5–17 years (not including schoolwork). Additionally, toddlers and preschoolers should not be allowed to stay stationary for more than one hour at a time, with the exception of time spent sleeping [1,34].

The American Academy of Pediatricians has also developed some recommendations that highlight the role of pediatricians, other physicians, schools, families, and government in assessing and improving the content of media and the security of the internet and children’s access to it [34]. The following are some actions pediatricians and other physicians can take:

1. They should educate themselves about current media and electronic devices, as well as keep up to date on their uses and influences on children.
2. Assess the patient’s home environment and exposure to media by asking screening questions about the allowed duration of time on electronic devices and whether there are any TVs or other electronic devices in the bedrooms. Any child who exhibits unusual behavior should be assessed for a more detailed history of electronic media exposure.
3. Advise parents to limit their children’s electronic media exposure with the following goals:
  - Try not to expose children younger than 2 years of age to electronic media at all, and limit screen time to less than 1 to 2 h per day for children older than 2.
  - Do not allow TV or electronic devices in children’s bedrooms.
  - Monitor the content of the internet and media that the children can access.
  - Set reasonable but firm rules regarding when and how long children may use electronic devices for.
  - Incorporate family time with electronic device/media exposure (for example, by watching a movie together).
  - Additionally, most importantly, be role models that follow and apply those rules as well.
4. Work with schools and educate them about the influence of media on children’s health. Encourage collaboration between teachers and parents to improve school and home environments and encourage innovative and educational use of the internet and electronic media.
5. Maintain communication with different health organizations and try to influence the entertainment industry to improve the content of their media and make it more pro-social and less violent.

In addition to the previous guidelines, it is worth mentioning a study that compared a customized 3D-printed collar with other cervical collars (Aspen Vista, Sport-aid) in 41 healthy young adults between the ages of 18 and 25. Head, neck, and trunk angles in three smartphone-using postures (standing, sitting with and without back support, and a neutral position). The personalized collar had a stronger posture-correcting impact than the Aspen Vista and Sport-aid collars [79], which could be another way to help these generations maintain a neutral position.

## 6. Conclusions

Preventing musculoskeletal (MSK) complaints caused by maladaptive postures requires attention to posture and attempts to maintain a neutral spine throughout everyday activities. TV, desktop computers, and heavy backpacks are linked to these maladaptive postures. However, the increasing use of portable gadgets, particularly smartphones, has increased the prevalence of MSK symptoms in adults and caused comparable symptoms and presentations in school-aged children and adolescents. Text neck syndrome is a clinical manifestation caused by handheld electronic device usage and the resultant text neck position. Headache, neck, and upper extremity pain, median or ulnar nerve compression, and psychological issues may occur. A correlation has been demonstrated between these maladaptive postures and major health issues, such as cervical spine disk prolapse and degenerative disk disease, later in life. However, a cause-and-effect relationship cannot be concluded. Given the frequency, long-term consequences, and disability burden of these musculoskeletal issues, it is crucial to act early, raise awareness, and provide preventative recommendations. With the ease of owning a cell phone, activities such as watching TV, playing video games, and using computers for business may now be carried out while sitting at a restaurant, riding a bus, or walking. Because these technologies are necessary for jobs and education, we cannot simply stop using them; therefore, we should focus on better methods to utilize these gadgets in a manner that allows children and adolescents to use them without harming their health or causing long-term issues. In summary, we should avoid utilizing these gadgets for lengthy periods with our bodies malpositioned. Physicians, parents, schools, and governments could raise awareness of the smartphone epidemic and implement new measures to address its health risks. Our paper’s take-home message is summarized by the following main points:

1. Maladaptive postures have been linked to musculoskeletal (MSK) problems. Maladaptive postures should thus be considered in children who do not have a clear underlying cause.
2. Multiple studies have found a correlation between early exposure to handheld electronics and MSK conditions such as DJD and disk prolapse later in life. Yet, unless an experimental study is conducted, a direct cause-and-effect relationship cannot be verified.
3. The accuracy of the present procedures used to certify cell phones by measuring their electromagnetic radiation levels requires further evaluation.
4. Most research has been conducted on young adults and college students. As a result, more research involving children and teenagers is required.
5. Improving classroom furniture is another issue that can be addressed, in addition to the guidelines intended to encourage people using electronic devices to maintain a neutral posture and exercise more.
6. It is crucial to conduct longitudinal cohort studies to assess the efficacy of the proposed preventative measures.

## Data Availability

Not applicable.
